# SMART Work Design: Accelerating the Diagnosis of Rare Diseases in the Western Australian Undiagnosed Diseases Program

**DOI:** 10.3389/fped.2020.00582

**Published:** 2020-09-18

**Authors:** Georgia J. Hay, Florian E. Klonek, Cati S. Thomas, Alicia Bauskis, Gareth Baynam, Sharon K. Parker

**Affiliations:** ^1^Center for Transformative Work Design, Future of Work Institute, Curtin University Graduate School of Business, Perth, WA, Australia; ^2^Business School, University of Western Australia, Perth, WA, Australia; ^3^Office of Population Health Genomics, Department of Health, Government of Western Australia, Perth, WA, Australia; ^4^Genetic Services of Western Australia, Department of Health, Government of Western Australia, Perth, WA, Australia; ^5^Western Australian Register of Developmental Anomalies, Department of Health, Government of Western Australia, Perth, WA, Australia; ^6^Faculty of Health and Medicine, Division of Pediatrics, University of Western Australia, Perth, WA, Australia; ^7^Telethon Kids Institute, University of Western Australia, Perth, WA, Australia; ^8^Faculty of Medicine, University of Notre Dame, Fremantle, WA, Australia; ^9^Faculty of Science and Engineering, Spatial Sciences, Curtin University, Perth, WA, Australia

**Keywords:** rare disease (RD), diagnos^*^, work redesign, teamwork, congenital abnomarlities, genomics

## Abstract

The accurate and efficient diagnosis of rare diseases, many of which include congenital anomalies, depends largely on the specialists who diagnose them – including their ability to work alongside specialists from other fields and to take full advantage of cutting-edge precision medicine technologies and precision public health approaches. However, highly specialized clinicians operating within a historically-siloed healthcare system is antithetical to the multi-disciplinary, collaborative, and creative approach that facilitates the diagnosis of rare diseases. The Western Australian Undiagnosed Diseases Program (UDP-WA) successfully re-designed the work of the involved clinicians to facilitate teamworking across silos. To understand the effectiveness of the Western Australian program, we draw on a SMART work design perspective (i.e., work that involves Stimulation, Mastery, Agency, Relations, and Tolerable demands). We propose that the redesign was successful in part because it improved crucial psychosocial work characteristics that are less prevalent in the broader work system, as identified in the SMART model. Based on the effectiveness of UDP-WA and its SMART design, we provide a framework that clinicians, healthcare managers, and policymakers can consider when they re-design work so that they can create SMART jobs within healthcare.

Individually, rare diseases are uncommon, each affecting fewer than 200,000 people in the U.S. (Orphan Drug Act of 1983). However, collectively, the ~10,000 different rare diseases will affect 1 in 12 people in their lifetime (1, 2)—which is an estimated 25–30 million Americans (3). Advances in genomic sequencing technology have facilitated a rapidly accumulating body of scientific knowledge on rare diseases, with new rare diseases appearing in the medical literature each week (4). Yet, despite these technological and scientific advancements, diagnosing which of the 10,000 rare diseases a given patient might have is an incredibly challenging task.

In addition to the vast amount of scientific literature on rare diseases, many rare diseases affect multiple physiological systems, thus requiring expertise from across multiple clinical specialties; this includes those with knowledge of congenital anomalies, as rare developmental defects during embryogenesis are the largest class of rare diseases (5). However, the structure of clinical knowledge within the health system is notoriously and increasingly siloed (6). Further, since ~70% of rare diseases have a genetic basis (7), clinical genetic expertise and expertise about advancements in (gen)omic testing technology is often required in the diagnosis of rare disease patients. In fact, genomic testing has recently been shown to be significantly more cost effective than traditional diagnostic approaches (8, 9)—yet, many clinicians lack this (gen)omic literacy, or the ability to access this expertise for their patients.

Consequently, and characteristically, patients with rare diseases embark on what is known as a “diagnostic odyssey,” whereby no single practitioner is looking at them “as a whole” (10). In Australia, ~30% of patients wait more than 5 years to reach a diagnosis (and 30% wait between 5 to upwards of 20 years), 30% of patients see more than six specialists, and 50% of patients receive at least one incorrect diagnosis (10). This “chaos that coexists with being undiagnosed” (11) often causes suffering, frustration, and uncertainty for patients and their families. It is also expected to be financially costly for the health system; just a *subset* of the patients with rare diseases (in the total Western Australian population of 2.5 million people) incur an estimated annual cost of AUD395 million to the Western Australian Health System in hospital admissions alone. In Western Australia, this subset represented 2% of the total population, yet accounted for 10.5% of hospital *inpatient* expenditure (12). While it is challenging to quantify exactly, it is likely that the resource-intensive nature of diagnosis bears a similarly disproportionate cost. It certainly bears a professional and emotional cost to clinicians, who must struggle against the siloed health system, an overload of clinical and scientific information, and a limited amount of time and resources to find answers for their patients—often for years, and often unsuccessfully.

## The Undiagnosed Diseases Program

In 2008, the United States' Undiagnosed Diseases Program (UDP-US) was conceived to address the unmet needs of individuals living with undiagnosed rare diseases. In the UDP, selected patients undergo a series of diagnostic tests and expert consultations, following which an interdisciplinary team of clinical and research experts examine the clinical and laboratory results for diagnostic clues. The cornerstone of the UDP concept is the use of *expert panel meetings*, in which the team of experts pool their expertise and discuss the patient's case in real time, generating creative ideas through a process of collaborative information processing and brainstorming. Thus, the UDP concept integrates previously siloed interdisciplinary expertise, and (gen)omic and other emerging precision medicine technologies, offering a disease-agnostic approach to diagnosis that capitalizes on the multi-disciplinary collaboration that is often necessary in the context of rare diseases. Starting as a single site at the National Institutes of Health (NIH), the NIH Common Fund initiative has since supported an expanded version of the program—the Undiagnosed Diseases Network—which now has 12 sites across the country.

Following the success of the UDP-US, and the identification of substantial patient need in Australia, the Western Australian UDP (UDP-WA) was implemented in March 2016 by a team of clinicians (from Genetic Services WA) and policymakers (from the Office of Population Health Genomics). While the UDP-WA functions with broadly the same structure as the UDP-US, it was implemented as a clinical service program within the WA public health system (13) to supplement existing genomic diagnostic work flows and multidisciplinary clinics (14)—whereas the UDP-US is supported by NIH research funding. After 1 year of operating, the percentage of families receiving a definitive diagnosis had nearly doubled, from 30% prior to the UDP-WA to 55% in the following year (14). The UDP-WA focuses on pediatric and pediatric-to-adult transition age groups; the majority of patients served by the UDP-WA have at least one congenital anomaly. For further detail on the UDP-WA please see Baynam et al. (13, 14).

Why is this approach so successful? We argue that a crucial part of the UDP's success is **good “work design**,” which fosters the psychological processes—social, motivational, and cognitive—for those engaging in the diagnostic work (i.e., the clinicians) that are necessary for diagnosing rare and complex diseases. For the past 3 years, we have been conducting research to *empirically* substantiate this claim [e.g., (15, 16)]. Here, we aim to introduce the theoretical background and conceptual basis of our new perspective on this program, and the interdisciplinary collaboration that occurs therein, which is rooted in organizational psychology. By drawing on the broader psychology literature, as well as our published research on the UDP (and quotes from this research), we aim to convince readers that the UDP is one example of how such a work design-focused perspective can elicit a deeper understanding of how to solve complex challenges in genetics and broader healthcare work. As research on work design spans a complex history of literature in the field of organizational psychology [e.g., see (17)], we rely on the SMART work design framework (18) to synthesize and convey our perspective, proposing that: *SMART work design could help to facilitate effective multi-disciplinary collaboration in healthcare*.

## Introducing: Work Design

*Work design* refers to “the content and organization of one's work tasks, activities, relationships, and responsibilities” (19). Issues in healthcare relating to team work, multiskilling, and the use of protocols in delivering patient care are all about work design because they are concerned with who does what, when, and how, in the work system. For example, poorly structured work in intensive care units was shown to predict high levels of workload which, in turn, was associated with lower perceived safety of patient care (20). While much of the technology and scientific knowledge that supports the delivery of patient care is rapidly advancing; historically, much less deliberate, evidence-based effort has been devoted to advancing the design of the work systems and structures that support healthcare workers in using these technologies [cf. (21)].

A great deal of theory and evidence in the organizational psychology literature has identified several key psychosocial “job characteristics” that define well-designed work, and that have been shown to lead to superior outcomes for both employees and organizations (17). The “SMART” model of good work design (18) synthesizes this literature into a simple framework. In what follows, we outline the core components of the SMART work design model—Stimulation, Mastery, Agency, Relations and Tolerable demands (see Table 1)—and use the UDP-WA as an example to illustrate the importance of well-designed work in interdisciplinary healthcare settings. All quotes are from Hay et al. (15).

**Table 1:** The SMART work design framework (18).

**Dimensions**	**Sub-dimensions**
Stimulating	• Adequate skill variety
	• Sufficient task variety
	• Problem solving demands
Mastery	• Role clarity
	• Feedback from others
	• Task identity
Agency	• Control over scheduling
	• Control over work methods
	• Decision making control
Relational	• Support from supervisor and peers
	• Sense of task significance
	• Perceived social worth
Tolerable demands	• Moderate time pressure and workload
	• Manageable emotional demands
	• Low role conflict

## SMART Work Design in the UDP-WA

The first important concept in the SMART model is that work should be “Stimulating”; jobs should involve a high degree of mental complexity and variety, including the opportunity to use a variety of skills (*skill variety*), engage in a wide variety of tasks (*task variety*), and to “think outside of the box” (*problem solving demands*). The UDP-WA cultivates a high level of stimulation, offering clinicians the opportunity to collaborate on cases that often fall outside their own specialty, thus also giving them the opportunity to develop new knowledge and skills related to other specialty areas (including genetics): “*… you just learn just by being there… I think it's such a good learning experience and so interesting.”(Pediatrician)*. Further, finding diagnoses for chronically-undiagnosed patients with a complex constellation of symptoms within the expert panel meetings requires engaging in a process of intensive problem solving, which many clinicians have described as highly stimulating: “…*like the art of medicine… I think everyone finds it really refreshing to actually do that, to have a real puzzle and to have to use all the medical knowledge that you've got to kind of piece it together.” (Pediatrician)*.

“Mastery” refers to the degree to which a job provides individuals with a sense of *role clarity* (e.g., about tasks and expectations), performance *feedback*, and *task identity* (i.e., completing a piece of work from beginning to end); and is an area in which the UDP-WA is particularly well-designed. Traditionally, clinicians who work with rare disease patients may receive very limited feedback; in some cases, they never receive updates about the outcomes of their clinical contribution, which generates a sense of chronic unease and uncertainty for the clinicians: “*Feedback is critical. Otherwise, it's like any human endeavor; if you're banging your head against the wall, it's always nice to know that there was some reason for it*.” *(Clinical Biochemist)*. Through the UDP-WA, clinicians receive frequent feedback about the outcomes of the diagnostic tests that were suggested by the team, and the impact of this on the progression toward a diagnosis for the patient.

Relatedly, members of the expert panel are able to follow the diagnostic process from beginning to end (i.e., they have high *task identity*). Prior to the implementation of the UDP-WA, this diagnostic process (and the ongoing patient care and management process more broadly) was marred by the tendency for rare disease patients to be treated as individual body systems: “*They'll see an endocrinologist for that problem, and they'll see a neurologist for that problem – but these two doctors don't talk to each other, so they don't get holistic treatment, because they're not seen as a whole person, they're seen as their individual body parts” (Policy officer)*. This is not only inefficient for the patient; it is also de-motivating and frustrating for the clinicians. Through the introduction of interdisciplinary panel meetings in the UDP-WA, clinicians are presented with a holistic picture of the patient, and the opportunity to see, and contribute to, the whole diagnostic process. This gives the clinicians a sense of task identity. Finally, in our continuous shadowing of the meetings over a 2 year period, we have observed that that the Program Director repeatedly reinforces psychological safety and reminds participants of all types and career stages to voice their ideas (even perceived “silly” ones) as they could potentially lead to a diagnosis. At the same time, clinicians are recruited into the expert panels based on their existing specialty role (which enhances their role clarity in this regard).

The third theme in the SMART model is “Agency,” which refers to the extent to which work provides employees with autonomy and decision-making opportunities —including, for example, deciding on one's work schedule (*work scheduling autonomy*) and which work methods to use (*work methods autonomy*). The UDP-WA offers the participating clinicians a substantial degree of autonomy with regards to their choice to attend the meeting (in person or via telehealth), as well as through offering them a variety of means through which they can choose to engage with the program (e.g., patient summaries, contributions via the digital health platform Patient Archive, e-mail comments). However, and perhaps more importantly, for clinicians who have an undiagnosed patient, the UDP-WA empowers them with the resources needed to diagnose their patient—thus increasing their work methods autonomy. Typically, the structure of the broader system (i.e., the typical structure of clinical work whereby clinicians have limited time with their patients, and typically little access to multidisciplinary diagnostic teams) inhibits clinicians' ability to get their patients the in-depth attention that they require; the availability of the UDP-WA empowers clinicians to give their patient the care they need, outside of the traditional confines of the health system (which, in the case of rare diseases, impedes the diagnostic process). Thus, while clinical work typically involves a high degree of autonomy to begin with, the empowering nature of the UDP-WA serves to increase this further by creating more options for clinicians.

The fourth concept that is crucial in the design of the UDP-WA is the opportunity to form relationships. Such “Relational” work is defined as the extent to which individuals experience *social support* (from supervisors and colleagues), *task significance* (i.e., a sense of purpose in relation to the lives of others and society more broadly), and *social worth* (i.e., the sense that their work is appreciated) in their role. Prior to the implementation of the UDP-WA, the opportunity to build relations between clinicians from different specialty areas was challenged by increasing siloing and fragmentation: “*a lot of the human interactions and the efficiencies of the stuff you used to do in the corridor when you walked past someone, didn't happen anymore*” (*Program Director*). The UDP-WA fosters positive team experiences, allows for opportunities to network with professionals outside of one's discipline, and provides the opportunity for multidisciplinary collaboration and support. While the work of clinicians is arguably already quite high on task significance, the way in which the UDP-WA streamlines and increases the efficiency of the diagnostic process serves to further amplify clinicians' perceptions that they are having a positive impact through their work: “*this collective real time thinking and investigating is the thing that gives it life” (Clinician)*.

Finally, “Tolerable demands” refers to the extent to which a job involves manageable levels of work demands, such as *time pressure and workload, emotional demands*, and *role conflict* (i.e., job feedback, instructions, and demands are inconsistent). The demands of clinical work are high—particularly in the context of ongoing and increasing institutional pressures to cut costs and “do more with less” [e.g., (22)]. However, the demands of finding diagnoses for patients with rare diseases are extraordinarily high; the sheer quantity of potential diagnoses, and the ever-increasing volume of scientific literature on such diseases, leads to incredible demands being placed on clinicians—who were previously facing such demands in relative isolation. The UDP-WA distributes the intellectual challenges and complexities of the diagnosis of rare diseases across the expert panel team, thus reducing the workload and emotional demands experienced by any one clinician; one clinician, in referring to their newly-diagnosed patient, said “that case has been weighing on my mind for *years*.” The UDP-WA also supports the team with the technical infrastructure and Nurse Coordinator support to alleviate the time burden associated with consolidating extensive clinical information. The role of the Nurse Coordinator in enabling teams to support rare disease patients is crucial: a recent study employing the Delphi method found a Nurse Coordinator to be the single most important factor in enabling a functional multidisciplinary team (23).

## Moving Forward

We argue that technological and scientific innovation—alone—are insufficient to address the challenges posed by rare disease diagnosis. Instead, further attention should be paid to the way in which the relevant tasks, activities, and work relationships are structured (i.e., *work design*) and the way in which this facilitates the collaborative, stimulating, and supportive environment necessary for clinicians to successfully diagnose rare diseases. This includes further documentation and investigation of the effects of SMART work design on key outcomes in healthcare. However, evidence thus far suggests that transformative work design is a critical element of sustainably addressing unmet needs and implementing precision medicine approaches for precision public health. Further, improving work structures is a critical element of sustainably implementing new (precision medicine) technologies, including through an equitable lens for those that need them the most, and for the most (i.e., precision public health). Thus, we encourage clinicians, managers, and policymakers who are working in the context of undiagnosed diseases—and other complex healthcare challenges that require interdisciplinary approaches—to proactively consider the role of SMART work in their organization. To this end, we also propose further widespread collaboration between people working in the healthcare sector and organizational psychologists, to ensure the design of a better system of healthcare work that is more effective, more engaging, and more equitable; for patients, employees, organizations, and economies.

## Web Resources

For more information about SMART work design, please visit: https://www.smartworkdesign.com.au/.

## Data Availability Statement

The original contributions presented in the study are included in the article/supplementary material, further inquiries can be directed to the corresponding author/s.

## Author Contributions

SP developed the SMART work design model (see transformativeworkdesign.com). GH and FK developed the conceptual material relating to SMART work design, with support and additional ideas from CT and SP. AB and GB contributed perspectives on healthcare policy and rare diseases, informing the application of the SMART work design concept to the specific context of rare disease diagnosis. CT wrote the first draft of the paper, with guidance from GH and FK. GH significantly refined the manuscript (through multiple iterations), with input from all other authors. All authors contributed to the conceptual basis of this paper.

## Conflict of Interest

The authors declare that the research was conducted in the absence of any commercial or financial relationships that could be construed as a potential conflict of interest.
